# Generation of Anti-Boa Immunoglobulin Antibodies for Serodiagnostic Applications, and Their Use to Detect Anti-Reptarenavirus Antibodies in Boa Constrictor

**DOI:** 10.1371/journal.pone.0158417

**Published:** 2016-06-29

**Authors:** Yegor Korzyukov, Udo Hetzel, Anja Kipar, Olli Vapalahti, Jussi Hepojoki

**Affiliations:** 1 University of Helsinki, Medicum, Department of Virology, Helsinki, Finland; 2 Institute of Veterinary Pathology, Vetsuisse Faculty, University of Zürich, Zürich, Switzerland; 3 University of Helsinki, Department of Veterinary Biosciences, Faculty of Veterinary Medicine, Helsinki, Finland; 4 University of Helsinki and Helsinki University Hospital, Department of Virology, Helsinki, Finland; Division of Clinical Research, UNITED STATES

## Abstract

Immunoglobulins (Igs), the key effectors of the adaptive immune system, mediate the specific recognition of foreign structures, i.e. antigens. In mammals, IgM production commonly precedes the production of IgG in the response to an infection. The reptilian counterpart of IgG is IgY, but the exact kinetics of the reptilian immune response are less well known. Boid inclusion body disease (BIBD), an often fatal disease of captive boas and pythons has been linked to reptarenavirus infection, and BIBD is believed to be immunosuppressive. However, so far, the study of the serological response towards reptarenaviruses in BIBD has been hampered by the lack of reagents. Thus we set up a purification protocol for boa constrictor IgY and IgM, which should also be applicable for other snake species. We used centrifugal filter units, poly ethylene glycol precipitation and gel permeation chromatography to purify and separate the IgM and IgY fractions from boa constrictor serum, which we further used to immunise rabbits. We affinity purified IgM and IgY specific reagents from the produced antiserum, and labelled the reagents with horseradish peroxidase. Finally, using the sera of snakes with known exposure to reptarenaviruses we demonstrated that the newly generated reagents can be utilised for serodiagnostic purposes, such as immunoblotting and immunofluorescent staining. To our knowledge, this is the first report to show reptarenavirus-specific antibodies in boa constrictors.

## Introduction

Immunoglobulins (Ig) play important roles in humoral immune responses against foreign antigens in vertebrates. In the majority of species they are comprised of heavy and light chains, which form hetero-oligomeric complexes linked by disulfide bonds [1]. Mammals have five heavy chain classes, α, δ, ε, γ and μ these give rise to IgG, IgA, IgE, IgD and IgM, respectively, by pairing with κ or λ light chains [1]. The humoral immunity, as judged by Ig genes, in ophidia (snakes) diversified approximately 300 million years ago [2] and shares some features with, but also differs from its mammalian counterpart [3]. In reptiles, as ectothermic animals, the immune response is directly affected by temperature [4], and the humoral immune response is slower than in mammals [3]. Moreover, similarly to mammals and birds, it is also known to be influenced by age, sex, and season and correlates with neuroendocrine rhythms [5]. While the antibody production in mammals reaches its maximum levels around 10–14 days after encountering an antigen, this can require up to 8 weeks in reptiles [6–11]. In mammals, the antibody production then declines within some weeks after reaching the peak [12]. In contrast, antibodies can persist in the blood for as long as 34 weeks after immunisation in reptiles [11] in which, however, the antibody titre does not increase upon the second encounter with the antigen [3]. Also, in contrast to mammals, only three Ig classes, IgY, IgD, and IgM, have been described in snakes [13]. In boids (*B*. *constrictor* and *C*. *hortulanus*), IgM, IgD and two classes of immunoglobulin IgY, IgYa and IgYb, are known [13]. IgY is also found in birds, which are phylogenetically closer to reptiles than mammals [14]. In both birds and reptiles, IgY is considered as the functional equivalent of IgG [15].

Boid inclusion body disease (BIBD) is an often fatal infectious disease of snakes, mainly affecting the *Pythonidae* and *Booidae* families [16]. The clinical signs of BIBD include regurgitation, head tremor, abnormal skin shedding, and neurological disturbances [16]. BIBD is also considered as immunosuppressive [17, 18], however, the immune response has so far not been studied in BIBD affected animals. There is strong evidence that the causative agents of BIBD are novel arenaviruses which have been identified in BIBD positive snakes by several research groups fairly recently [19, 20, 21]. The identification of these novel viruses led to the establishment of a new genus, *Reptarenavirus*, within the family *Arenaviridae* [22]. Arenaviruses have a bisegmented negative-sense RNA genome with ambisense coding strategy [23]. The L segment encodes the RNA-independent RNA polymerase (RdRp) and the Z protein (ZP), whereas the glycoprotein precursor (GPC) and the nucleoprotein (NP) are encoded in the S segment [24–26]. The pathognomonic intracytoplasmic inclusion bodies (IB) seen in BIBD [15, 16] mainly consist of reptarenavirus NP [19, 20]. However, the absence of other viral proteins in the IB has not yet been confirmed. While the coincidence of reptarenaviruses and BIBD suggests an aetiologic relationship, the experimental evidence is still lacking. Also, very recently, we and an American group reported that snakes with BIBD are often co-infected with multiple reptarenaviruses [27, 28].

In this study we established a protocol for the purification of IgY and IgM from snake serum, and used the purified Igs to generate anti-boa IgY and IgM (referred to as anti-IgM and anti-IgY) antibodies. We used affinity purification to limit the cross reactivity between the generated reagents, and labelled the resulting reagents with horseradish peroxidase. Using sera from BIBD positive snakes and recombinant reptarenavirus antigens, we then demonstrated that the newly generated reagents can be used in serodiagnostic assays. Furthermore, we gathered evidence that only a proportion of snakes with BIBD generate anti-reptarenavirus antibodies.

## Materials and Methods

### Polyethylene Glycol (PEG) Precipitation of Immunoglobulins

A pool of serum from two boa constrictors snakes (Table 1: snakes #8 and #9; corresponding to snakes 11 and 28 of [20]), (V = 1.5 ml) was diluted to 15 ml with phosphate-buffered saline (PBS) and concentrated to approximately 7.5 ml using a 100 kDa cutoff centrifugal filter device (Millipore). The remaining supernatant was diluted to 15 ml with PBS and concentrated to approximately 4 ml as above, followed by one additional dilution-concentration cycle. The remaining supernatant (V = 4 ml) was cleared by centrifugation (16,000 x g, 5 min, 4°C), the supernatant diluted once more to 15 ml with PBS, and concentrated as above to a final volume of 1.8 ml. To precipitate the proteins, 5.4 ml of precipitation solution A (4.7% PEG 6000, 25 mM, 75 mM NaCl, pH 7.5) was added to the mixture and the resulting solution was incubated for 5 min at room temperature (RT) before pelleting the proteins by centrifugation (10,000 x g, 10 min, 4°C). After centrifugation the pellet was stored on ice, and 2.3 ml of precipitation solution B (37.5% PEG 6000, 25 mM, 75 mM NaCl, pH 8.0) was added to the supernatant. After mixing, the resulting solution was centrifuged (10,000 x g, 10 min, 4°C), the pellet stored on ice, and 1.3 ml of precipitation solution B added to the supernatant, mixed and centrifuged (10,000 x g, 10 min, 4°C). The resulting supernatant and pellet were stored on ice until analysis.

**Table 1:** Detection of anti-UHV NP antibodies in snakes with BIBD. The sera were collected from *Boa constrictors* with BIBD as characterised in [20].

Snake number*	IgM antibodies (WB/IF)	IgY antibodies (WB/IF)	IHC	Tissue (antigen by WB)	RT-PCR	Blood smear	Age
#1 (8)	-/-	-/-	+	+	+	+	Juvenile
#2 (9)	-/-	-/-	+	+	+	-	Juvenile
#3 (35)	-/-	-/-	-	-	-	+	Juvenile
#4 (5)	-/-	-/-	+	+	+	+	Juvenile
#6 (7)	-/-	-/-	+	+	+	+	Juvenile
#7 (10)	+/+	+/+	+	+	+	-	Juvenile
#8 (11)	-/-	-/-	+	+	-	+	Adult
#9 (28)	-/-	+/+	-	-	-	?	Adult

* Numbers #1–9 refer to numbering of Fig 8 from Hetzel et al., 2013, and the numbers in parentheses refer to the original animal numbers in Table 1 from Hetzel et al., 2013. IHC = immunohistochemistry, WB = western blot, IF = immunofluorescence.

### Gel Permeation Chromatography

The final pellet resulting from the PEG precipitation was solubilized in Tris-buffered saline (TBS, 50 mM Tris, 150 mM NaCl, pH 8.0), loaded onto a Superdex 200 HR (GE Healthcare Lifesciences) 10/30 column (V = 24 ml), equilibrated into PBS (pH 7.4), and the proteins were separated at a flow rate of 0.4 ml/min. The fractions containing IgM and IgY were pooled and further purified with a Sephacryl S-200 HR 16/60 column (V = 120 ml) at a flow rate of 1 ml/min using PBS as the eluent. The collected fractions were analyzed by sodium dodecyl sulfate-polyacrylamide gel electrophoresis (SDS-PAGE) and the fractions containing the purified protein were pooled and concentrated using a 30 kDa cut off centrifugal filter device (Millipore).

### Immunisation, Antibody Purification and Labelling

Purified IgM and IgY were used for the immunisation of rabbits by a commercial provider (BioGenes GmbH; www.biogenes.de) applying the following scheme: pre-bleeding and immunisation (150 μg, day 0), booster #1 (70 μg, day 7), booster #2 (70 μg, day 14), first bleeding and booster #3 (150 μg, day 28), booster #4 (70 μg day 49). Two rabbits were used and immunised with purified IgM and IgY, respectively. For purification of anti-IgM and anti-IgY antibodies, affinity columns were prepared by coupling the purified proteins onto CnBr-activated Sepharose 4B (GE Healthcare Lifesciences) according to the manufacturer’s protocol. The coupled matrices were packed into disposable PD-10 columns (GE Healthcare Lifesciences) and equilibrated with PBS. The anti-IgM antiserum was initially passed through an IgY affinity column, followed by purification of the flow through using an IgM affinity column. Similarly, the anti-IgY antiserum was passed through an IgM affinity column prior to purification with an IgY affinity column. The affinity purifications were performed by gravity flow. Briefly, after passing the antiserum through, the column was washed with 15 column volumes (CV) of PBS, the bound proteins were eluted using 0.1 M Glycine (pH 2.5), and the fractions neutralised by adding 2 M Tris-HCl, pH 8.5. The affinity purified anti-IgM and anti-IgY antibodies were dialysed against PBS using 10K cut off Slide-A-Lyser cassettes (ThermoFisher Scientific), concentrated using a 30 kDa cut off centrifugal filter device (Millipore), and horseradish peroxidase (HRP) labelled with the EZ-link Activated Peroxidase Antibody Labeling Kit (ThermoFisher Scientific), following the manufacturer’s protocol. The labelled antibodies were purified from free HRP using HiTrap Protein G Sepharose Fast Flow (GE Healthcare Lifesciences) columns according to the manufacturer’s protocol.

His-tagged recombinant University of Helsinki Virus (UHV-1) Z protein (rZP) was cloned, produced, and purified as described for recombinant UHV-1 NP (29). Purified rZP was coupled to CnBr-activated Sepharose 4B (GE Healthcare Lifesciences) following the manufacturer’s protocol. The rZP affinity column was used to affinity purify the ZP binding fraction from antiserum produced against inactivated UHV-1 [20] similar to the above described for anti-IgM and -IgY affinity purification.

### SDS-PAGE and Immunoblotting

Proteins were separated by SDS-PAGE under reducing or non-reducing conditions. For immunoblotting, the proteins separated on SDS-PAGE were transferred onto nitrocellulose (Whatman) by wet blotting. The protein concentrations were determined using the bicinchoninic acid (BCA) protein assay kit (ThermoFisher Scientific) according to the manufacturer’s instructions. The recombinant NP and its N—and C-terminal fragments (rNP, rNP-N, and rNP-C) of University of Helsinki virus-1 (UHV-1) described in [29] were used as the antigen (1:1:1 pool, 1.5 μg of total protein per lane) when measuring anti-reptarenavirus antibodies by immunoblotting. The immunoblotting with snake sera in brief: The nitrocellulose membranes were blocked (3% skimmed milk and 1% bovine serum albumin [BSA] in TBS with 0.05% Tween-20 [T-TBS]) on an orbital shaker for 30 min at RT, incubated overnight at 4°C with snake sera (diluted (1:300) in T-TBS with 3% BSA), washed three times with T-TBS, incubated for 1 h at RT with either anti-IgM or -IgY antibody (both 1:2,000 dilution in blocking buffer), washed three times with T-TBS, incubated for 1 h at RT with IRDye 800CW-labelled donkey anti-rabbit (dilution 1:10,000 in blocking buffer, LI-COR Biosciences), and washed three times with T-TBS and once with PBS prior to recording the results, using an Odyssey Infrared Imaging System (LI-COR Biosciences). The immunoblottings with IgY and IgM as the antigens were done following a similar procedure, except that enhanced chemiluminescence with in-house reagents and recording on X-ray film (FUJI Medical RX) was used for recording the results with HRP-labeled anti-IgM and –IgY.

### Mass Spectrometry

Mass spectrometry was performed by the Proteomics Unit, Institute of Biotechnology, University of Helsinki. Briefly, silver stained [30] protein bands were cut out of the polyacrylamide gel and “in-gel” digested, the cystein bonds were reduced with 0.045M dithiothreitol (Sigma-Aldrich) for 20 min at 37°C and alkylated with 0.1M iodoacetamide (Sigma-Aldrich) at RT. Samples were digested by adding 0.75 μg trypsin (Sequencing Grade Modified Trypsin, Promega), and after digestion the peptides were purified with C18 microspin columns (Harvard Apparatus) according to manufacturer’s protocol. The dried peptides were reconstituted in 30 μl of buffer A (0.1% trifluoroacetic acid, TFA, in 1% acetonitrile, ACN).

Liquid chromatography coupled to tandem mass spectrometry (LC-MS/MS) analysis was carried out on an EASY- nLC1000 (Thermo Fisher Scientific) connected to a Q Exactive mass spectrometer using the Xcalibur version 2.2 SP 1.48 (Thermo Fisher Scientific). The LC-MS/MS samples were separated using a two-column set-up consisting of a 2 cm C18 trap column (Thermo Fisher Scientific), followed by a 15 cm C18 analytical column (Thermo Fisher Scientific). The linear separation gradient consisted of 5% buffer B in 5 min from 5% to 35% buffer B in 60 min, 80% buffer B (0,1% TFA acid in 98% ACN) in 5 min and 100% buffer B in 10 min at a flow rate of 0.3 μl/min. 5 μl of sample was injected per LC-MS/MS run and analysed. Full MS scan was acquired with a resolution of 70,000 at normal mass range in the Orbitrap analyzer. The method was set to fragment the 10 most intense precursor ions with HCD (energy 28).

Acquired MS2 scans were searched against a home-made snake Ig database (genes in [31], including python Igs GenBank: AFR33768.1, AFR33767.1, AFR33766.1, AFR33765.1, and AFR33764.1) using the Sequest search algorithms in a Thermo Proteome Discoverer. Allowed mass error for the precursor ions was 15 ppm, and 0.8 Da for the fragments. A static residue modification parameter was set for carbamidomethyl +57.021 Da of cysteine residue. Methionine oxidation was set as dynamic modification +15.995 Da. Only full-tryptic peptides were allowed for scoring, maximum of 2 missed cleavages were considered.

### Viruses, Cell Culture, and Immunofluorescence Staining

Vero E6 cells were grown (5% CO_2_, 37°C) on pre-sterilised glass coverslips in minimal essential medium (MEM) (Sigma-Aldrich) supplemented with 10% foetal bovine serum (FBS), 2 mM L-glutamine, 100 IU/ml penicillin, and 100 μg/ml streptomycin, pH 7.2 to 7.3. When the cells reached 80–90% confluency, the media was replaced with media containing 30 mM HEPES and cells were inoculated with Vero E6 adapted UHV-1 [20] and incubated for 2 d at 30°C. At 2 days post infection (dpi) the media was removed and the cells were fixed with 4% paraformaldehyde (pH 7.4) for 15 min, washed with PBS, permeabilised for 10 min (0.3% Triton-X-100 and 3% BSA in PBS), and stored in PBS prior to probing. The immunofluorescence staining in brief: The fixed and permeabilised cells were incubated with affinity purified protein specific antibodies (anti-NP [29] and anti-ZP, produced against UHV-1), polyclonal anti-UHV-1 antibody [20], or snake serum (Table 1: snakes #6, #7 and #9), at respective dilutions of 1:500, 1:500, 1:250, and 1:300 in PBS for 2 h at RT, washed three times with PBS, then incubated (this step only when using snake serum) for 1 h with anti-IgM and anti-IgY (1:2,000 dilution in PBS) at RT, washed three times with PBS, incubated for 45 min with AF488 goat anti-rabbit (1:1,000 dilution in PBS, Life Technologies), washed three times with PBS and twice with water, dried, and the coverslips were mounted onto glass slides.

## Results and Discussion

### Enrichment of Immunoglobulins from Snake Serum

**Fig 1:**
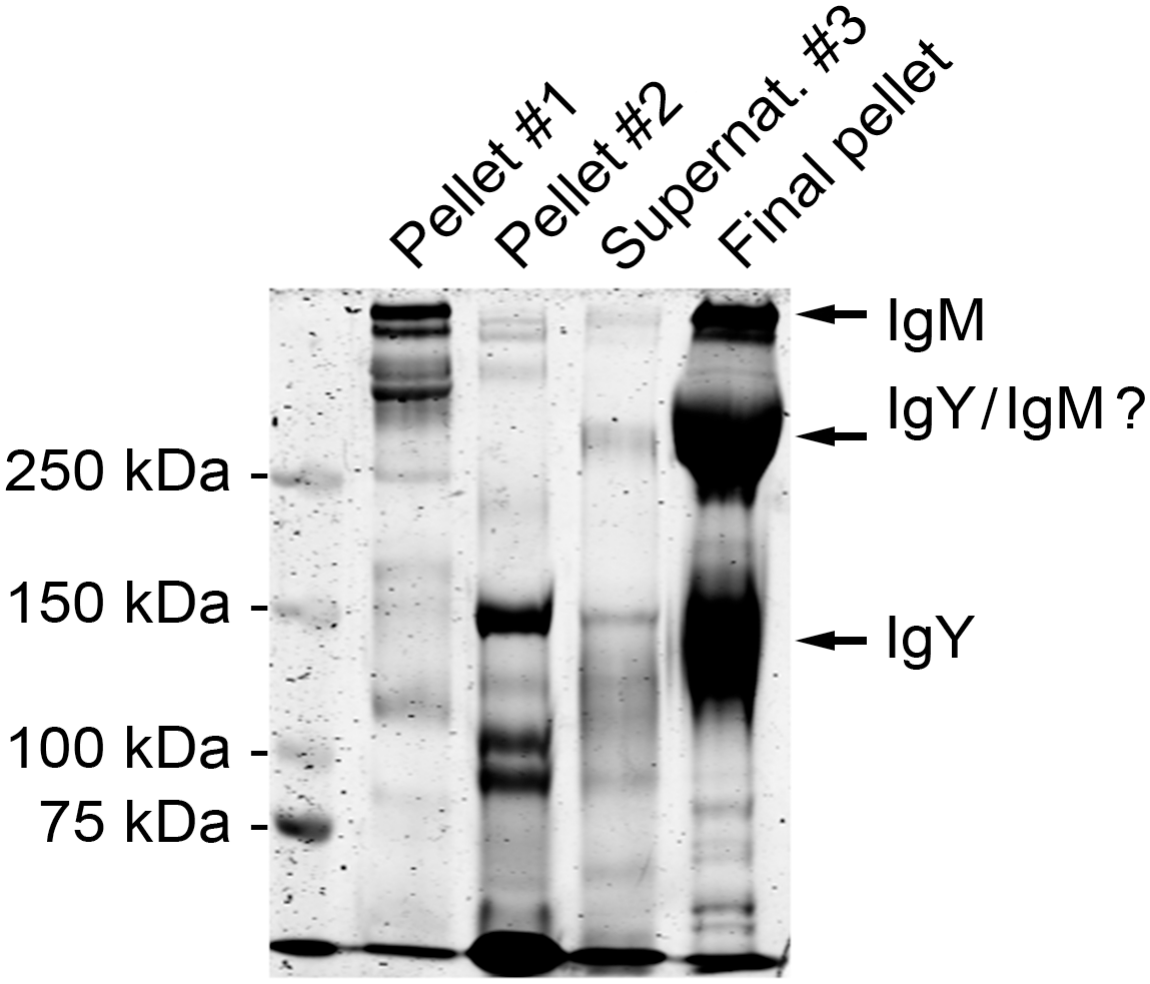
Polyethylene glycol (PEG) precipitation of proteins from *B*. *constrictor* serum. The serum proteins were initially transferred into PBS using a 100 kDa cut off centrifugal filter device, and precipitation was achieved by step-wise addition of PEG followed by centrifugation. The pelleted proteins were analysed by SDS-PAGE under non-reducing conditions. The left lane shows the molecular weight marker (Precision Plus Protein Dual Color, Bio-Rad), the second lane shows protein precipitated at ~3.5% PEG (pellet #1), the third lane shows protein precipitated at ~12% PEG (supernatant #2), the fourth lane shows protein in supernatant after the second pelleting (supernatant #3), and the fifth lane shows proteins precipitated at ~15% PEG (final pellet).

There are several protocols in the literature describing the precipitation of avian immunoglobulins from egg yolk [32, 33], and we reasoned that a similar approach might be applicable for the enrichment of Igs from snake serum. After an initial dilution in PBS, we concentrated the serum using a 100 kDa cut off filter to transfer the proteins into PBS and to eliminate smaller proteins. We performed protein precipitation by step-wise increasing the PEG concentration and subsequent centrifugation. SDS-PAGE analysis of the pelleted material showed that the final pellet contained proteins with a mobility consistent with the size of IgM and IgY (Fig 1).

### Separation of IgY and IgM by Gel Permeation Chromatography

**Fig 2:**
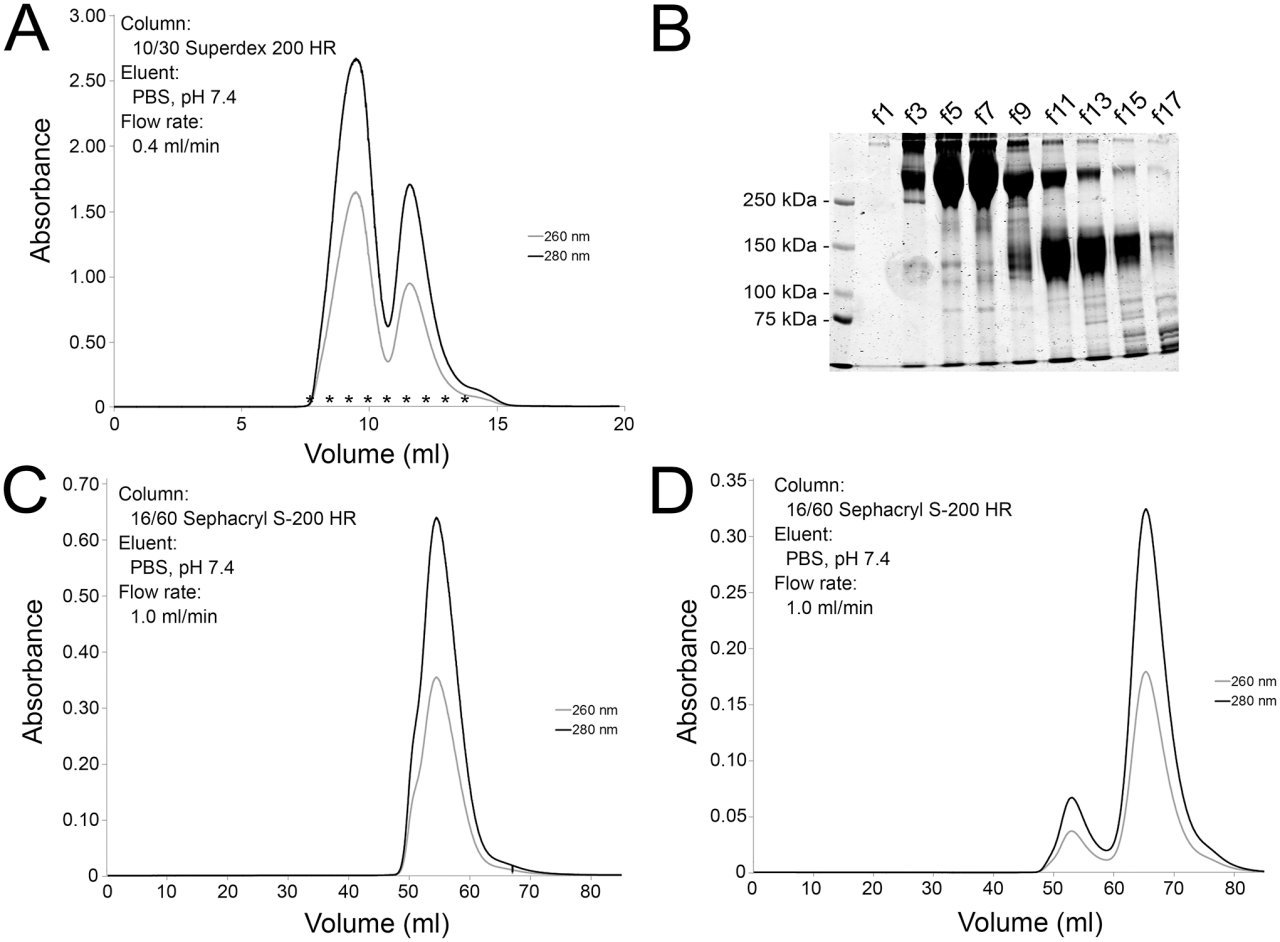
Purification of IgM and IgY using gel permeation chromatography. **A)** The IgM and IgY containing pellet from the PEG precipitation was loaded onto a 10/30 Superdex 200HR column (GE Healthcare) and the proteins were eluted with PBS at a flow rate of 0.4 ml/min. Dual wave length absorbance monitoring, A_260nm_ and A_280nm_, was used for protein detection. **B)** Fractions collected during the gel permeation chromatography were analysed by SDS-PAGE under non-reducing conditions, the left lane shows molecular weight marker (Precision Plus Protein Dual Color, Bio-Rad) and the subsequent lanes labelled f1-f17 represent the fractions (marked with * in the chromatogram). **C)** The fractions (the left “peak” in A, corresponding to f1-f9 in B) containing IgM were pooled, concentrated using a 100 kDa centrifugal filter (Millipore), and loaded onto a 16/60 Sephacryl S-200HR column (GE Healthcare). The proteins were eluted with PBS at flow rate of 1.0 ml/min, and dual wave length absorbance monitoring, A_260nm_ and A_280nm_, was used for protein detection. **D)** The fractions (the second “peak” in A, corresponding to f10-f17 in B) containing IgY were pooled, concentrated using a 100 kDa centrifugal filter (Millipore), and loaded onto a 16/60 Sephacryl S-200HR column (GE Healthcare). The proteins were eluted with PBS at a flow rate of 1.0 ml/min, and dual wave length absorbance monitoring, A_260nm_ and A_280nm_, was used for protein detection.

**Fig 3:**
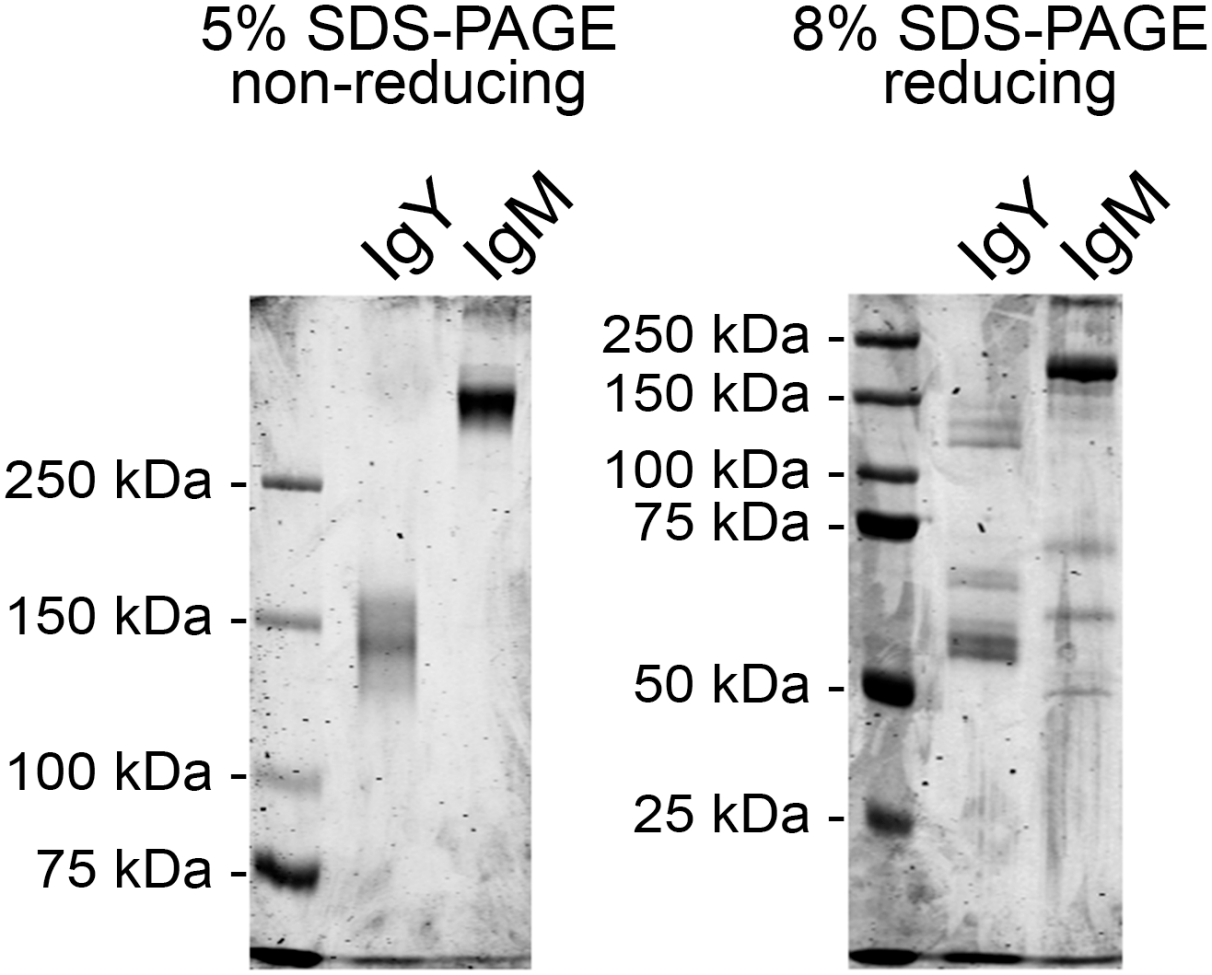
SDS-PAGE analysis of the purified IgM and IgY. The IgM and IgY containing fractions were concentrated using a 100 kDa cut off centrifugal filter (Millipore), and the protein composition determined by SDS-PAGE. The left panel shows proteins separated and visualised using Coomassie staining on a 5% SDS-PAGE under non-reducing conditions, the right panel shows proteins separated on a 8% SDS-PAGE under reducing conditions. The left lane in both gels shows the molecular weight marker (Precision Plus Protein Dual Color, Bio-Rad).

The final pellet obtained by PEG precipitation contained at least two major protein fractions, IgM and IgY, and thus we utilised gel permeation chromatography to separate the proteins by size. We loaded the final pellet onto a 10/30 Superdex 200HR column, eluted the proteins with PBS (Fig 2A), and analysed the protein containing fractions by SDS-PAGE (Fig 2B). The elution profile showed two major protein fractions that partially overlapped. Thus we pooled the two major protein “peaks” and performed gel permeation chromatography for each pool. For the second gel permeation we used a 16/60 Sephacryl S-200HR column for increased resolution. The IgM (Fig 2C) and IgY (Fig 2D) fractions were adequately separated in the second chromatography step and we therefore pooled the main “peaks” for subsequent analyses. The two fractions were separated by SDS-PAGE under non-reducing and reducing conditions. Under non-reducing conditions, the IgY fraction produced two bands of approximately 140 and 160 kDa (by mobility), whereas IgM produced a strong band of approximately 500 kDa (by extrapolation) and a faint band barely migrating in the separation gel (Fig 3, left panel). Under reducing conditions, the IgY fraction produced bands of approximately 140, 130, 120, 70, and 60 kDa by mobility (Fig 3, right panel). The 60 and 70 kDa bands likely represent the IgY heavy chain [31], accordingly, the 120–140 kDa bands may represent homodimers of the heavy chain. A light chain was not detected. Considering a previous report which found the amount of light chain in IgY1 and IgY2 of *Python molurus bivitattus* to be very low in comparison to the heavy chain [31], it is likely that it was also below the detection limit in the boa sera (Fig 3). The IgM fraction produced a multiplicity of bands under reducing conditions, the major bands corresponding to approximate sizes of 175, 70, 60, and 50 kDa. In the *Python molurus bivitattus*, the size of the IgM heavy chain is approximately 65 kDa without, and 70–90 kDa with glycosylation [31], thus the observed bands at 50–70 kDa likely represent the IgM heavy chain. The prominent band at 170 kDa might represent an incompletely dissociated IgM molecule (Fig 3). The IgY and IgM fractions were further analysed by trypsin digestion and LC-MS/MS. The mass spectrometry yielded three peptides matching *Python bivitattus* IgY for the purified IgY and a single peptide matching *Orthriophis taeniurus* IgD for the IgM. The protein identification relies on sequence databases, and since the sequences for boa constrictor IgY and IgM are not available, the low number of identified peptides is not surprising. The identification of peptides matching python IgY strongly suggests that our IgY fraction is actually the IgY of boa constrictor. Based on the SDS-PAGE analysis and the fact that we used serum as the source of Igs (IgD is mostly membrane-bound at least in mammals), it seems less likely that the IgM fraction would actually represent IgD. Instead, we rather think that the mass spectrometry confirms the IgM fraction to also represent Ig, most likely IgM based on the other analyses.

### Generation and Affinity Purification of Anti-IgY and Anti-IgM Antibodies

**Fig 4:**
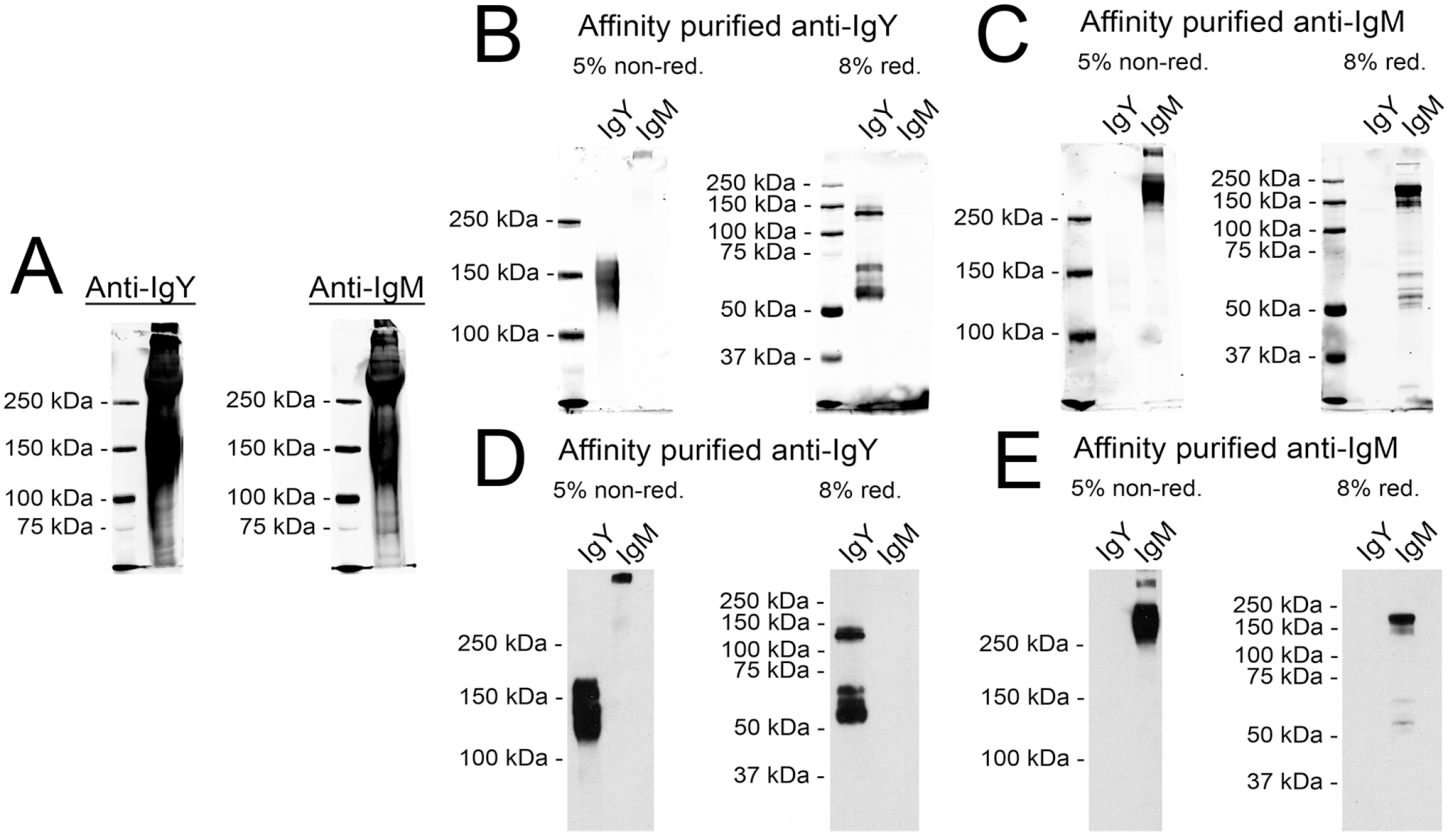
Characterisation and purification of the antisera against IgM and IgY. **A)** The antisera produced by immunizing rabbits with either purified IgY (Anti-IgY) or IgM (Anti-IgM) were used for immunoblotting the proteins present in the final pellet of the PEG precipitation (see lane 5 in Fig 1 for reference). The left lane in both gels shows the molecular weight marker (Precision Plus Protein Dual Color, Bio-Rad). The results were recorded using Odyssey infrared imaging system (LI-COR Biosciences). **B)** Immunoblots with affinity purified anti-IgY. The left lanes show the molecular weight marker (Precision Plus Protein Dual Color, Bio-Rad), the middle lanes represent the purified IgY fraction, and the right lanes the purified IgM fraction (see Fig 3 for IgY and IgM reference). The proteins were separated on both 5% SDS-PAGE under non-reducing and 8% SDS-PAGE under reducing conditions. The results were recorded using Odyssey infrared imaging system (LI-COR Biosciences). **C)** Immunoblots with affinity purified anti-IgM. The lanes and the detection are as in **B**. **D)** Immunoblots with affinity purified and HRP labelled anti-IgY. The left lanes represent the purified IgY fraction, and the right lanes the purified IgM fraction. The proteins were separated on both 5% SDS-PAGE under non-reducing and 8% SDS-PAGE under reducing conditions. The marker is not visible, since the results were recorded on X-ray film using enhanced chemiluminescence. **E)** Immunoblots with affinity purified and HRP labelled anti-IgM. The lanes and the detection are as in **D**.

Antibodies against the putative IgM and IgY fractions were produced by immunising one rabbit with each antigen. The antisera were tested by immunoblotting with the final protein pellet containing both IgY and IgM of Fig 1 (Fig 4A). Since both immunoblots seemed almost identical, indicating considerable cross reactivity between the two antisera, we proceeded with affinity purification. We generated affinity columns by coupling the IgY and IgM fractions separately to CnBr activated Sepharose (GE Healthcare). The produced anti-IgY antiserum was initially passed through an IgM-coupled column to remove potentially cross reacting antibodies, then the IgY-binding fraction was purified using an IgY-coupled column. Finally, the IgG fraction was purified from the bound protein with a protein G column. The anti-IgM antiserum underwent a similar purification scheme. The resulting purified IgG fractions were tested in immunoblots, using the purified IgY and IgM as test antigens; this showed only minimal remaining cross reactivity (Fig 4B and 4C). To aid the further use of the generated secondary antibodies, we coupled the IgG fractions with horseradish peroxidase (HRP), and evaluated the reagents using the purified IgY and IgM as test antigens (Fig 4D and 4E). Both reagents also showed good performance in enhanced chemiluminescence (ECL) detection, and only the anti-IgY antibody exhibited mild cross reactivity with IgM under non-reducing conditions.

### Detection of Snake IgM and IgY by Immunoblotting and Immunofluorescence Assay

**Fig 5:**
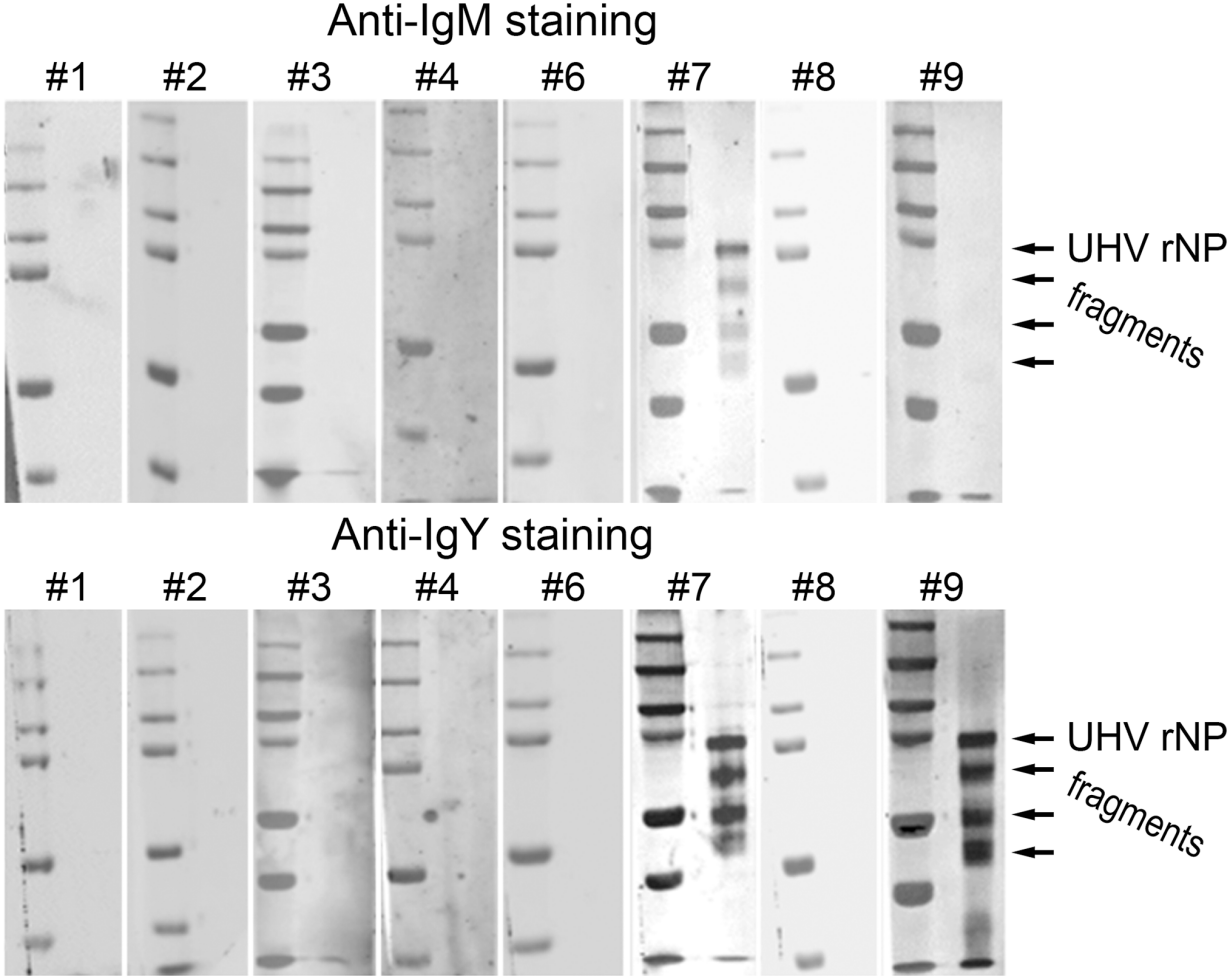
Screening of sera from *Boa constrictors* with BIBD for antibodies against reptarenaviruses by immunoblotting. Protein pools of Ni-NTA affinity purified recombinant UHV NP (the full-length protein, and N- and C-terminal fragments) separated by SDS-PAGE under reducing conditions were used as the antigens. The membranes were probed with snake serum, and detection of binding was done using either anti-IgY or anti-IgM in combination with an IRDye800 labelled secondary antibody. The immunoblot signals were recorded with Odyssey infrared imaging system (LI-COR Biosciences). The snake serum (or blood) samples #1–4 to #6–9 correspond to the animal numbers of our earlier study (see Table 1, and [20]). The left lanes show the molecular weight marker (Precision Plus Protein Dual Color, Bio-Rad). The arrows indicate the size of the recombinant proteins.

Our main motivation for the production of anti-boa Ig reagents was to study the immune response against reptarenaviruses in snakes with BIBD. The immune response or the lack of it is of particular interest, since it might play a role in the pathogenesis of BIBD. We have recently identified several reptarenaviruses [20, 28] in snakes with BIBD and have shown that the antiserum against the NP of one reptarenavirus (UHV-1) broadly cross reacts with various reptarenavirus species. In our first report characterising UHV-1, we showed evidence of anti-reptarenavirus antibodies in some snakes with BIBD, using an indirect enzyme-linked immunosorbent assay, ELISA [20]. Also, in a more recent study we produced and purified recombinant NP (rNP) of University of Helsinki virus (UHV-1) and its N- and C-terminal fragments (rNP-N and rNP-C) [29]. Thus, to test the newly generated anti-boa Ig reagents for their applicability in the detection of an antibody response in snakes, we used a pool of the above mentioned recombinant proteins as the test antigens for immunoblotting with snake serum. We chose to use the previously studied serum samples (except for #5 of the original samples, which we had run out of) for the immunoblots, since the initial results had suggested at least two of these to be antibody positive (Table 1). By immunoblotting antibodies against reptarenavirus NP were detected in 2 of the 8 snakes (#7 and #9) (Fig 5), both of which had yielded strongest inhibition in the indirect ELISA [20]. Interestingly, snake #7 possessed both IgM and IgY antibodies, whereas snake #9 was only positive for IgY antibodies.

**Fig 6:**
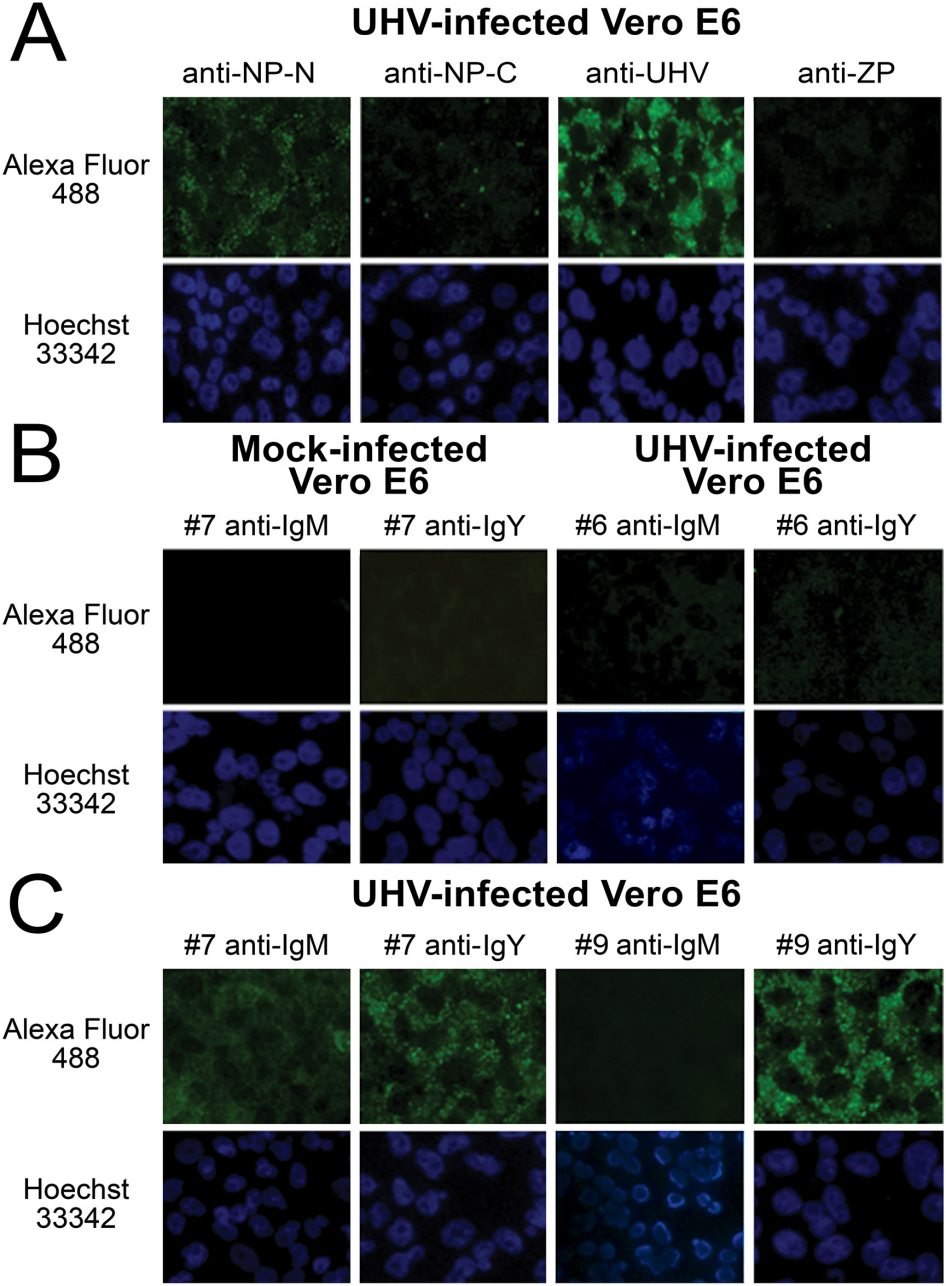
Immunofluorescence staining of reptarenavirus infected cells using protein specific antibodies and snake sera. **A)** Vero E6 cells infected with UHV adapted to grow in these cells were fixed at 2 dpi and stained with antibodies against NP (anti-NP-N and anti-NP-C), Z protein (anti-ZP), UHV (anti-UHV). **B)** The left panels: mock infected Vero E6 cells stained with snake serum #7, which was found positive for both IgM and IgY in immunoblot (see Fig 5), the panels on right: Vero E6 cells infected with UHV (as in **A**) and stained with snake serum #6, which was found negative for both IgM and IgY in immunoblot (see Fig 5). **C)** Vero E6 cells infected with UHV (as in **A**) stained with snake sera #7 (both IgM and IgY positive in immunoblot, Fig 5) and #9 (IgM negative and IgY positive in immunoblot, Fig 5). The antibody binding was detected using both anti-IgY and anti-IgM antibodies and AlexaFluor488 conjugated donkey anti-rabbit immunoglobulins (Molecular Probes). Nuclei were visualized using Hoechst 33342.

To evaluate whether the generated anti-boa Ig reagents could be utilised in immunofluorescence (IF) staining, we infected Vero E6 cells with a reptarenavirus adapted to this cell line [20], and stained the infected cells using the boa sera #1–4 and #6–9 with either anti-IgM or anti-IgY. We also stained the infected cells with antibodies against NP-N, NP-C, ZP, and inactivated UHV-1 to demonstrate the cellular expression patterns for the structural proteins (Fig 6A). The staining with seronegative samples did not yield substantial background fluorescence (two representatives presented in Fig 6B), whereas the sera of snakes #7 and #9 (positive in the immunoblots) yielded matching IF signals (Fig 6C). The anti-NP staining was represented by a predominantly granular fluorescence pattern, while the anti-ZP protein staining resulted in a weak, finely granular reaction (Fig 6A). The antiserum against inactivated UHV showed a combination of diffuse and granular staining, in which the GPC accounts for the diffuse staining (Fig 6A). By comparing the staining patterns of protein specific antibodies and anti-UHV to the staining patterns obtained with the snake sera, it is obvious that the strongest reaction is against the NP (Fig 6C). However, the staining of the infected cells with snake sera and anti-IgM antibody resulted in a more diffuse fluorescence pattern which may indicate that snakes possess antibodies also against at least the GPC. Additional experiments with the generation and application of recombinant reptarenavirus GPC (comprising the spike-forming SSP-GP1-GP2 complex) or purified viruses would allow to more accurately determine against which protein(s) the antibody responses are generated.

The results of our serological study, albeit with relatively few samples, indicate that only relatively few snakes with confirmed BIBD possessed anti-reptarenavirus antibodies (Table 1). Also, of the two snakes with anti-reptarenavirus antibodies (#7 and #9), BIBD was confirmed only in #7. Snake #9 had been co-housed for 6 months with a BIBD positive snake and was found to be reptarenavirus infected, but had not developed BIBD. Unfortunately, neither the time of exposure to reptarenaviruses nor the time between onset of clinical signs and sampling is known for the other snakes. BIBD is considered as immunosuppressive, as affected snakes often develop secondary infections and neoplasms [17, 18]. The lack of serum antibodies in a large proportion of snakes with BIBD could be explained by immunosuppressive properties of reptarenaviruses. The ZP and NP of rodent-borne mammarenaviruses are known to interfere with interferon production and the innate immune system [34, 35, 36]. Studies with a prototypic arenavirus, lymphocytic choriomeningitis virus (LCMV), have shown strain-related differences in the ability to prevent a virus-specific immune response [37]. Furthermore, experimental studies provided evidence that neonatal or *in utero* infection with LCMV results in the deprivation of freely circulating antibodies against the virus [38]. It is also possible that reptarenaviruses somehow interfere with the antibody production, for example by infecting B-cells, plasma cells, or the progenitor cells of the bone marrow. Furthermore, it cannot be excluded that the antibody titres had declined below detectable levels during prolonged infection. We recently observed, when studying the viruses isolated from snakes #1, #2, #4, and #8 by next-generation sequencing that these snakes were actually co-infected with two or more reptarenavirus species [28]. Sequential infection by multiple reptarenaviruses could be amplified by antibody-dependent enhancement, which could then result in the progression towards BIBD. Alternatively, the snakes could become immune tolerant to reptarenaviruses when the infection occurred during the foetal phase or prior to the development of immune competence. For example in the desert tortoise, *Gopherus agassizii*, the immune competence only develops 4–6 months after hatching [5]. Whether the immune system of other reptiles, such as snakes, develops within a similar time scale, is so far not known. We hope to answer some of the above mentioned questions in future studies.

## Concluding Remarks

Serodiagnostics in snakes have so far been hampered by the lack of suitable diagnostic tools. In this study we described a protocol for the purification of Igs from *B*. *constrictor* serum. We used the purified IgM and IgY to produce secondary antibodies, by combining affinity purification and horseradish peroxidase labelling. We further tested the generated reagents to study the IgM and IgY responses towards reptarenaviruses in boa constrictors utilising a pre-characterized sample panel. We were able to demonstrate that the reagents are applicable in both immunoblotting and immunofluorescence staining. Although it seems that the reagents we generated cannot be used for BIBD diagnostics, they may prove valuable tools for the detection of antibodies against some other infectious agents of snakes. Furthermore, the HRP labelled reagents should enable the setup of serodiagnostics in an ELISA format. To fully utilise the generated reagents, it will be essential to determine the snake species, the Igs of which can be recognised by the reagents.

Even after the recent advances in the characterisation of reptarenaviruses, the diagnosis of BIBD still mainly relies on the clinical exclusion of other diseases and the detection of the typical inclusion bodies in cells in blood smears and histological specimens. The high genetic variation among reptarenaviruses greatly hampers the diagnosis by RT-PCR, and based on our results BIBD diagnosis should not be attempted by serology either. Thus far it seems that the detection of reptarenavirus antigen might provide the most sensitive tool for the diagnosis of BIBD.
